# Supplementary material for: The Association of Work Satisfaction and Burnout Risk in Endoscopy Nursing Staff—A Cross-Sectional Study Using Canonical Correlation Analysis
Source: Int J Environ Res Public Health. 2020 Apr 24;17(8):2964. doi: 10.3390/ijerph17082964 (PMC7215598; doi:10.3390/ijerph17082964)
Supplement: Supplementary file 1 [file ijerph-17-02964-s001.pdf]

Supplementary Material

Supplementary Table S1. Factor intercorrelations between factors of the Work Satisfaction Questionnaire in the confirmatory factor analysis

	colleagues	professional development	payment	supervisor	general job satisfaction
occupational function	.41	.44	.13	.33	.53
colleagues		.38	.14	.47	.58
professional development			.33	.45	.63
payment				.26	.45
supervisor					.65

Supplementary Table S2. Standardised canonical coefficients and structure correlations of the second predictor canonical variate

predictor canonical variate	standard canonical coefficients	structure correlations
occupational function	.72	.58
colleagues	.58	.41
professional development	.28	.18
payment	-.11	-.26
supervisor	-.14	-.10
general job satisfaction	-.89	-.28

Supplementary Table S3. Standardized canonical coefficients and structure correlations of the second criterion canonical variate

criterion canonical variate	standard. canonical coefficients	structure correlations
emotional exhaustion	1.14	.49
personal accomplishment	0.64	.50
depersonalization	-0.72	-.16

Supplementary Table S4. Main results of commonality analysis for the second prediction canonical variate.

predictor canonical variate	coefficient	% total
unique to occupational function	.041	39.4
unique to general job satisfaction	.040	38.5
unique to colleagues	.025	23.7
unique to personal rewards		
common to occupational function and general job satisfaction	-.0145	-14.1
common to colleagues and general job satisfaction	-.0125	-12.1
